# Chaetocin enhances dendritic cell function via the induction of heat shock protein and cancer testis antigens in myeloma cells

**DOI:** 10.18632/oncotarget.17517

**Published:** 2017-04-29

**Authors:** Manh-Cuong Vo, Thanh-Nhan Nguyen-Pham, Hyun-Ju Lee, Sung-Hoon Jung, Nu-Ri Choi, My-Dung Hoang, Hyeoung-Joon Kim, Je-Jung Lee

**Affiliations:** ^1^ Research Center for Cancer Immunotherapy, Chonnam National University Hwasun Hospital, Hwasun, Jeollanamdo, Republic of Korea; ^2^ Department of Hematology-Oncology, Chonnam National University Hwasun Hospital, Hwasun, Jeollanamdo, Republic of Korea

**Keywords:** chaetocin, dendritic cells, multiple myeloma

## Abstract

Dendritic cells (DC)-based vaccines are considered useful in cancer immuno-therapy, and the interactions of DC and dying tumor cells are important and promising for cancer immunotherapy. We investigated whether chaetocin could be used to induce death of myeloma cells, for loading onto DCs can affect DCs function. In this study, we show that the dying myeloma cells treated with chaetocin resulted in the induction of heat shock protein (HSP) 90, which was inhibited by antioxidant N-acetyl cysteine, and showed an increase in the expression of MAGE-A3 and MAGE-C1/CT7. DCs loaded with chaetocin-treated dying myeloma cells produced low levels of IL-10 and enhanced the cross presentation of DCs. Additionally, these DCs most potently inhibited regulatory T cells, induced Th1 polarization and activated myeloma-specific cytotoxic T lymphocytes compared with DCs loaded with UVB-irradiated dying myeloma cells. These results suggest that the pretreatment of myeloma cells with chaetocin can enhance DC function through the up-regulation of HSP90 and cancer testis antigens in dying myeloma cells and can potently induce the Th1 polarization of DCs and myeloma-specific cytotoxic T lymphocytes.

## INTRODUCTION

Multiple myeloma (MM) is a clonal B-cell malignancy that is characterized by the accumulation and proliferation of plasma cells in the bone marrow [1, 2]; Recently the use of novel anti-myeloma drugs, such as immune modulatory drugs [pomalidomide and lenalidomide] and proteasome inhibitors (bortezomib) has improved disease outcome and extended the overall survival time, however MM has remained an incurable in most patients [3–5], thus, there is a need for another treatment approach can be taken to induce MM rejection. Immune-based therapeutic options that use antigen-presenting cells (APCs) which increased potency are considered an attractive tool in cancer immunotherapy [6–9]. Dendritic cells (DCs) the most potent APCs, play a crucial role in antitumor immunity via the generation of cytotoxic T lymphocytes (CTLs) [10–19]. Therefore, DC-based immunotherapy is expected to be effective in treatment of MM patients. More recent studies demonstrated improved clinical response by DC-based immunoglobulin idiotype vaccination for MM patients [20, 21]. However, few clinical results are yet available, and a strategy to improve the effect of DCs vaccination in MM patients is required. An important consideration to improve the efficacy of DC vaccination in MM patients is the sources of effective tumor antigens, including myeloma cell lysates, myeloma apoptotic bodies, DC–myeloma cell hybrids, or DC transfected with myeloma-derived RNA, instead of using idiotype proteins with weak antigenicity [22–25].

Most of chemotherapy agents kill cancer cells through the induction of apoptosis. However, not all agents have capacity to induce immunogenic cell death [26–30], therefore, studies of the specific anticancer agents that lead to apoptosis and immunogeneic cancer cell death, and whether these processes can enhance antitumor immunity against cancer are required.

Chaetocin is a small-molecule thiodioxopiperazine natural product produced by Chaetomium species fungi [31–33], that is currently in development as a candidate antimyeloma agent, and has potent *in vitro* and *in vivo* activity shown by its ability to impose increased levels of cellular oxidative stress [34]. Chaetocin has also been found to be useful as a histone methyl-transferase inhibitor, with interest in whether the compound is sufficient to kill various cancer cells [35].

In this study, we investigated whether chaetocin could be used to induce death of tumor cells, for loading onto DCs to enhance myeloma-specific antitumor immune responses. Here, we show that chaetocin-induced dying myeloma cells can be used as a source of tumor antigens for loading onto DCs, which could elicit potent anti-myeloma activity of cytotoxic T lymphocytes (CTLs) due to the expression of heat shock proteins (HSPs) and cancer testis antigens (CTAs) on dying myeloma cells, as a mechanism of the immunogenic cell death of MM cells.

## RESULTS

### Expression of HSP90 and CTAs in dying myeloma cells

**Figure 1:**
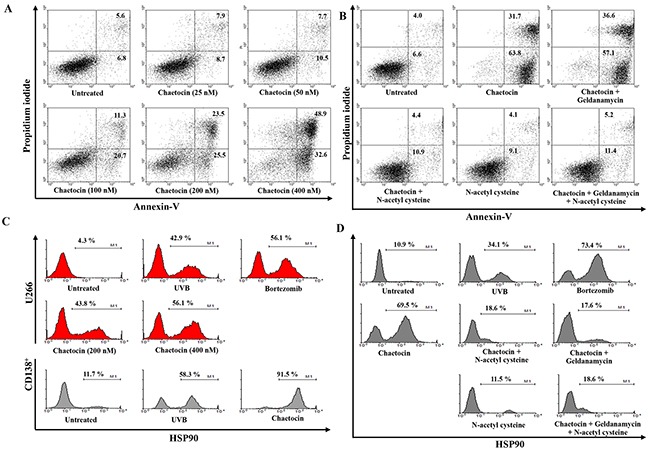
Chaetocin produces the highest expression of HSP90, MAGE-A3, and MAGE-C1/CT7 in dying myeloma cells **(A)** The percentage of dying myeloma cells after treatment with chaetocin was analyzed using Annexin-V/Propidium iodide staining. Treatment with 400 nM chaetocin showed a significant increase in the population of dying U266 cells compared with other groups (∼82% of cells underwent apoptosis). **(B)** The population of dying U266 myeloma cells treated with 400 nM chaetocin was not inhibited by pretreatment with the 10 nM geldanamycin but it is inhibited by pretreatment with the antioxidant agent 10 mM N-acetyl cysteine. **(C)** The HSP90 expression in dying myeloma cells after treatment with UVB irradiation, 100 nM bortezomib, or 400 nM chaetocin was analyzed by flow cytometry. The dying myeloma cells (U266 cell lines and CD138^+^ cells from patients) treated with 400 nM chaetocin showed higher expression of HSP90 compared with untreated myeloma cells or UVB-irradiated myeloma cells. **(D)** The levels of HSP90 expression in dying U266 myeloma cells treated with 400 nM chaetocin were decreased by pretreatment with 10 mM N-acetyl cysteine or 10 nM geldanamycin.

**Figure 2:**
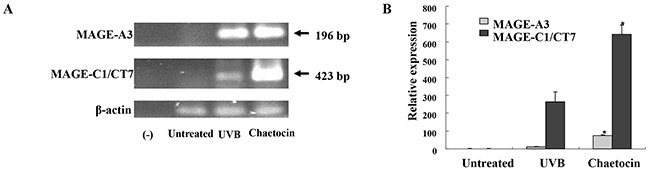
Chaetocin produces the highest expression of MAGE-A3, and MAGE-C1/CT7 in dying myeloma cells The expression levels of the cancer testis antigens, such as MAGE-A3 and MAGE-C1/CT7, in dying U266 cells were evaluated by qRT-PCR **(A)** and the samples were separated by agarose gel electrophoresis and visualized by ethidium bromide staining **(B)**. Dying U266 cells treated with 400 nM chaetocin showed a significant increase in the expression of MAGE-A3 and MAGE-C1/CT7 compared with the untreated myeloma cells or UVB-irradiated U266 cells (*, *P* < 0.05). Data are representative of more than three experiments.

To induce dying U266 myeloma cells, U266 cells were treated with chaetocin in a dose-dependent manner (25 to 400 nM). The population of dying cells after 24 h of treatment was analyzed by Annexin-V/PI staining. Treatment with 400 nM chaetocin showed a significant increase in the population of dying U266 cells compared with the other groups (∼82% of cells underwent apoptosis) (Figure 1A). The population of dying U266 myeloma cells treated with 400 nM chaetocin was not inhibited by pretreatment with the 10 nM geldanamycin (Biomol *Enzo Life Sciences*, Farmingdale, NY, USA) but it is inhibited by pretreatment with the antioxidant agent 10 mM N-acetyl cysteine (Sigma-Aldrich, St. Louis, MO, USA; Figure 1B). The data suggest that chaetocin could effectively induce dying U266 myeloma cells through reactive oxygen species production. To evaluate the expression of HSP90 in the dying myeloma cells, the dying myeloma cells were stained with HSP90-FITC-labeled mAb (BD Bioscience, San Jose, CA, USA) and analyzed by flow cytometry. The dying myeloma cells (U266 cell lines and CD138^+^ cells from patients) treated with 400 nM chaetocin and 100 nM Bortezomib (Sigma-Aldrich) showed higher expression of HSP90 than untreated myeloma cells or UVB-irradiated myeloma cells (Figure 1C) or isotype control (Supplementary Figure 1). In addition, the levels of HSP90 expression in dying U266 myeloma cells treated with chaetocin were inhibited by pretreatment with 10 mM N-acetyl cysteine or 10 nM geldanamycin (Figure 1D). These results indicated that chaetocin might act to increase the expression of HSP90 in response to cellular stress on dying myeloma cells. To provide direct evident for the expression level of CTAs, such as MAGE-A3 and MAGE-C1/CT7 in dying U266 myeloma cells, qRT-PCR was performed before the products were analysed on a 1% agarose gel. Dying U266 myeloma cells treated with 400 nM chaetocin showed a significant increase in the expression of MAGE-A3 and MAGE-C1/CT7 compare with untreated myeloma cells or UVB-irradiated myeloma cells (Figure 2A and 2B).

### Characteristics of DCs loaded with dying myeloma cells

**Figure 3:**
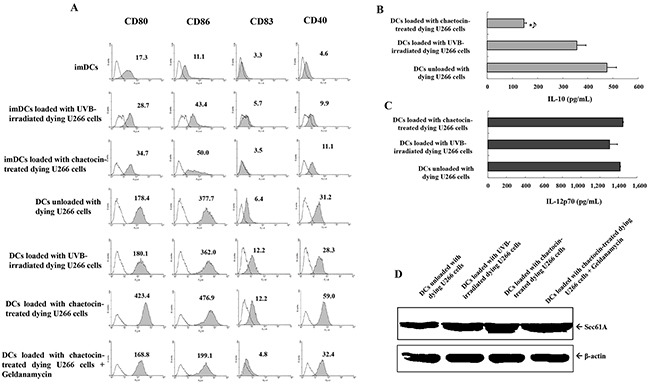
Characterization of dendritic cells (DCs) loaded with dying U266 cells **(A)** The phenotype of DCs was analyzed for the expression levels of CD80, CD86, CD83, and CD40 using flow cytometry. DCs loaded with chaetocin-treated dying U266 cells showed the increased expression of maturation molecules compared with imDCs, imDCs loaded with UVB-irradiated dying U266 cells, imDCs loaded with chaetocin-treated dying U266 cells, DCs unloaded with dying U266 cells, or DCs loaded with UVB-irradiated dying U266 cells, and the expression of maturation molecules on DCs loaded with chaetocin-treated dying U266 cells was decreased by the addition of geldanamycin. Representative histograms show the marker expression levels (shaded) compared with those of the isotype controls (black line). Enzyme-linked immunosorbent assay (ELISA) revealed that DCs loaded with dying U266 cells treated with chaetocin showed a significant decrease in the production levels of IL-10 **(B)**, while the levels of IL-12p70 **(C)** production were not significantly different than those of DCs unloaded with dying U266 cells, or DCs loaded with dying U266 cells treated with UVB irradiation (*, *P* < 0.05). The data are provided as means (pg/mL) ± standard deviation (SD) of triplicate cultures from three independent experiments. **(D)** The expression of Sec61A on DCs loaded with dying U266 cells was evaluated by Western blotting. DCs loaded with chaetocin-treated dying U266 cells showed the increased expression of Sec61A compared with DCs unloaded with dying U266 cells, and DCs loaded with UVB-irradiated dying U266 cells, and the expression of Sec61A on DCs loaded with chaetocin-treated dying U266 cells was partially decreased by the addition of geldanamycin. Data are representative of three independent experiments.

To generate DCs maturation, immature DCs (imDCs) were activated by LPS for another 2 days, and dying U266 myeloma cells were added 2 hours after the addition of LPS. DCs loaded with chaetocin-treated dying U266 cells showed increased expression of maturation molecules CD80, CD86, CD83 and CD40 compared with imDCs, imDCs loaded with UVB-irradiated dying U266 cells, imDCs loaded with chaetocin-treated dying U266 cells, DCs unloaded with dying U266 cells, or DCs loaded with UVB-irradiated dying U266 cells and the expression of maturation molecules on DCs loaded with chaetocin-treated dying U266 cells was decreased by the addition of geldanamycin (Figure 3A). The levels of the IL-12p70 and IL-10 cytokines of DCs loading with dying U266 cells were measured after subsequent CD40L stimulation. DCs loaded with chaetocin-treated dying U266 cells showed significantly decreased production of IL-10 compared with DCs unloaded with dying U266 cells, or DCs loaded with UVB-irradiated dying U266 cells (Figure 3B). However, IL-12p70 production did not affect DCs (Figure 3C). The expression level of Sec61A, an endoplasmic reticulum translocon protein related to cross presentation in DCs, in DCs unloaded with dying U266 cells and DCs loaded with chaetocin-treated or UVB-irradiated dying U266 cells was evaluated by Western blotting. DCs loaded with chaetocin-treated dying U266 cells showed increased expression of Sec61A compared with DCs unloaded with dying U266 cells, and DCs loaded with UVB-irradiated dying U266 cells, and the expression of Sec61A on DCs loaded with chaetocin-treated dying U266 cells was partially decreased by the addition of geldanamycin (Figure 3D). These results indicated that DCs loaded with chaetocin-treated dying U266 cells might act to enhance the expression of maturation phenotypes and produce low levels of the inhibitory cytokine IL-10 and to increase cross presentation.

### Enhancement of CTLs and the Th1 polarization and inhibition of CD25^+^Foxp3^+^ T cells by DCs loaded with chaetocin-treated dying U266 cells

**Figure 4:**
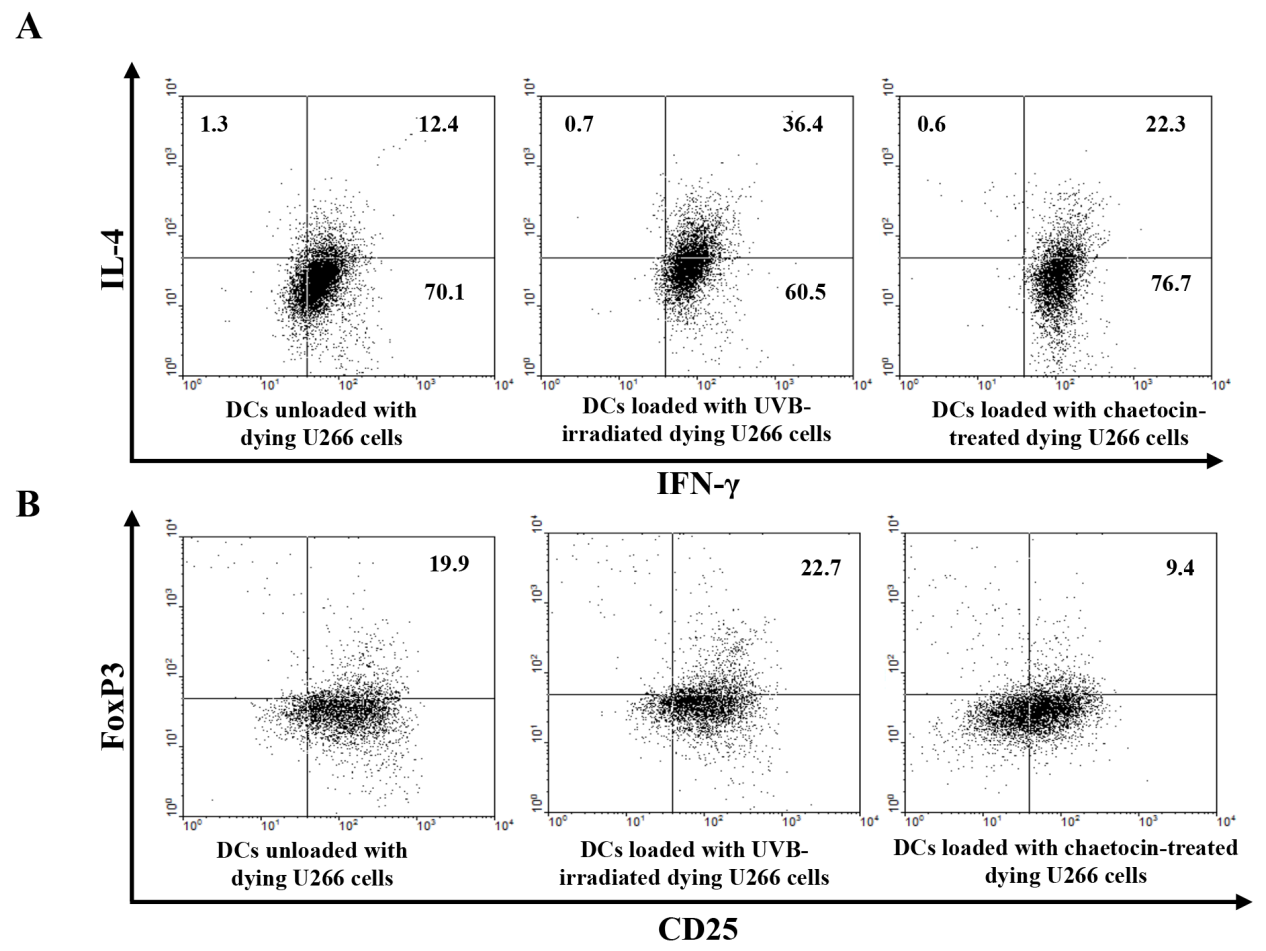
Induction of Th1 polarization and inhibition of CD4^+^CD25^+^Foxp3^+^ T cells by DCs loaded with dying U266 cells treated with chaetocin **(A)** The intracellular IFN-γ and IL-4 levels were measured using flow cytometry. DCs loaded with dying U266 cells treated with chaetocin exhibited the highest Th1 response (IL-4^−^IFN-γ^+^) up to 77% of the total T cells. **(B)** The proportions of CD25^+^Foxp3^+^ T cells were measured by flow cytometry. The percentage of CD25^+^Foxp3^+^ T cells showed a decrease in CD4^+^-naïve T cells, induced by DCs loaded with dying U266 cells treated with chaetocin, compared with DCs unloaded with dying U266 cells, or DCs loaded with dying U266 cells treated with UVB irradiation. Data are representative of more than three experiments.

To investigate the effect of chaetocin-treated dying myeloma cells on the induced antitumor response against myeloma cells, we evaluated the T cell polarization capacity of CD4^+^-naïve T cells by stimulation of the DCs. Allogeneic CD4^+^-naïve T cells stimulated with DCs loaded with chaetocin-treated dying U266 cells showed the increased production of IFN-γ and the decreased production of IL-4 compared with those stimulated with DCs unloaded with dying U266 cells, or DCs loaded with UVB-irradiated dying U266 cells (Figure 4A). In addition, the induction of CD25^+^Foxp3^+^ T cells was decreased in DCs loaded with chaetocin-treated dying U266 cells compared with DCs unloaded with dying U266 cells, or DCs loaded with UVB-irradiated dying U266 cells (Figure 4B). These data indicated that chaetocin-treated dying myeloma cells have an immunomodulating effect to promote the Th1 polarization of naïve T cells and suppress CD25^+^ Foxp3^+^ T cells indirectly by DCs in MM.

**Figure 5:**
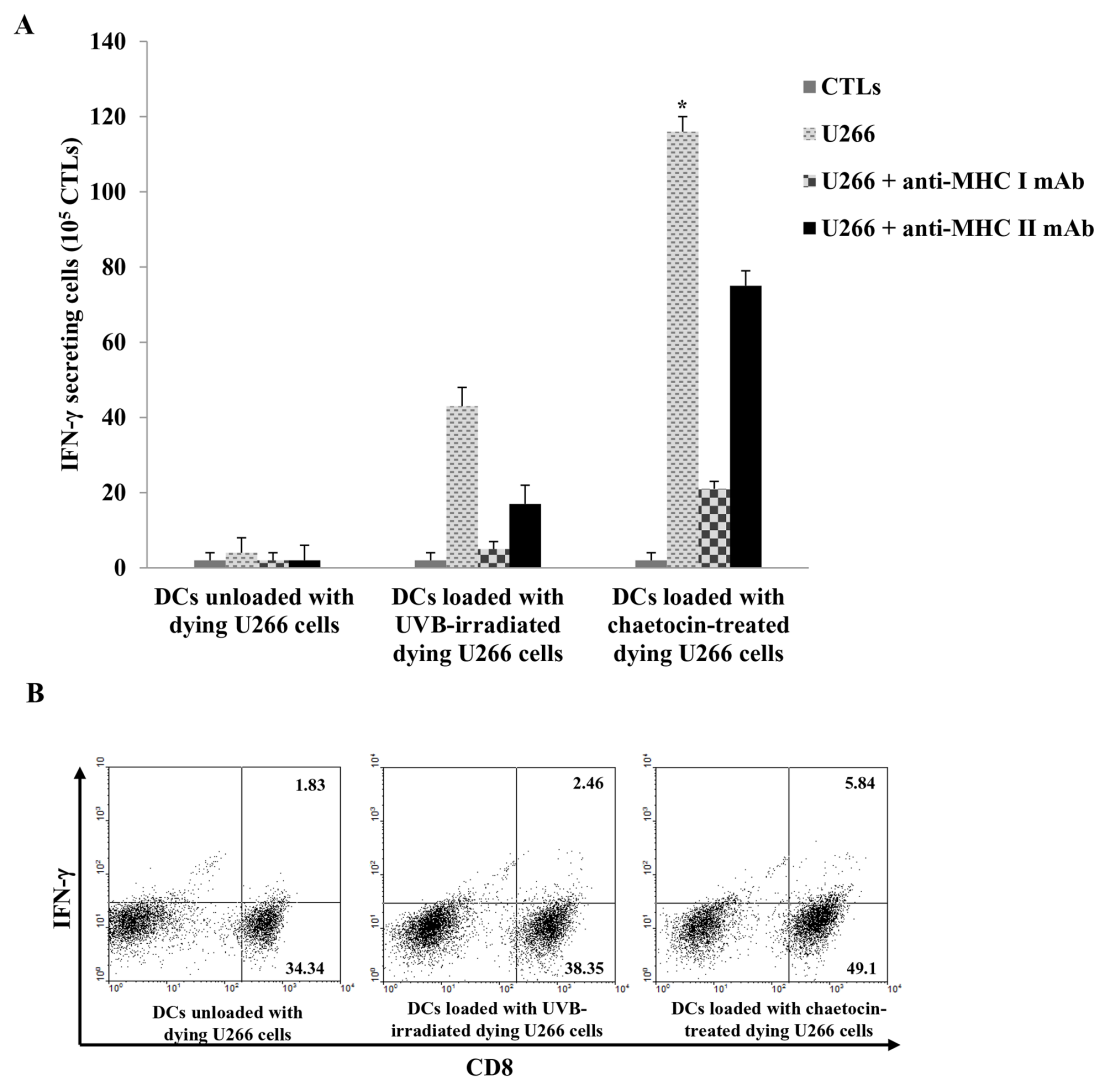
Activation of cytotoxic T lymphocytes(CTLs) by DCs loaded with dying U266 cells treated with chaetocin Autologous T cells (1 × 10^6^ cells) were stimulated by autologous DCs (2 × 10^5^ cells) loaded with dying U266 myeloma cells; the interferon (IFN)-γ enzyme-linked immunospot (ELISPOT) assay and intracellular staining were performed to quantify IFN-γ production by CTLs **(A)** CTLs, generated by DCs loaded with dying U266 cells treated with chaetocin, induced significantly increased IFN-γ production compared with DCs unloaded with dying U266 cells, or DCs loaded with dying U266 cells treated with UVB irradiation (*,*P* < 0.05). **(B)** Intracellular staining of IFN-γ production in CD8^+^ T cells, activated by DCs loaded with dying U266 cells treated with chaetocin, was higher than DCs unloaded with dying U266 cells, and DCs loaded with dying U266 cells treated with UVB irradiation. Data are representative of more than three experiments.

To investigate the anti-myeloma effect of CTLs generated by the DCs loaded with dying myeloma cells, IFN-γ production by CTLs was measured by the ELISPOT assay and intracellular staining. CTLs stimulated with DCs loaded with chaetocin-treated dying U266 cells displayed a greater number of IFN-γ production against U266 myeloma target cells than those stimulated by DCs unloaded with dying U266 cells, or DCs loaded with UVB-irradiated dying U266 cells (*, *P* < 0.05) (Figure 5A & 5B). Blocking studies using anti-MHC I and II antibodies showed a specific response of CTLs against U266 target cells, which were MHC class I-restricted and partly MHC class II-related. In addition, DCs loaded with chaetocin-treated dying U266 cells led to the increased proportion of CD8^+^ T cells (Supplementary Figure 2A) and a decreased proportion of CD4^+^ T cells compared with DCs unloaded with dying U266 cells, or DCs loaded with UVB-irradiated dying U266 cells (Supplementary Figure 2B).

## DISCUSSION

Although several disadvantages related to DCs vaccine have been reported, DC-based vaccines are still a promising tool as alternative approaches to prolong survival of patients with incurable MM [2]. However, tumor antigen source is still a challenge for clinical setting and the development of appropriate tumor antigen sources is crucial point for the successful clinical setting [20, 36].

In this study, we demonstrated that, the dying myeloma cells treated with chaetocin could be used a potential tumor antigen source for DCs in MM treatment. We have shown that chaetocin is able to potently induce myeloma cells apoptosis, and elicits the expression of heat shock protein (HSP) 90 and cancer testis antigens (CTA) in myeloma cells. Moreover, we found that pretreatment of myeloma cells with chaetocin enhanced DCs functions through inhibiting the production of IL-10 and enhancing the cross presentation of DCs. Additionally, these DCs most potently inhibited regulatory T cells, induced Th1 polarization and activated myeloma-specific cytotoxic T lymphocytes. These data demonstrated that chaetocin-induced apoptosis leads to an enhanced autologous antitumor T cell response to primary human tumor cells without the need for an additional stimulus.

Prior studies have documented that chaetocin appears to represent a promising agent for antimyeloma therapy with *in vitro* and *in vivo* activity mediated by the imposition of oxidative stress [37]. Furthermore, the expression of HSPs is increased dramatically in response to cellular stress [38]. In addition, chaetocin was found to be a specific histone methytransferase (HMT) inhibitor [35], HMT inhibitor is a type of epigenetic modulatory drug, that plays a critical role in epigenetic regulation of cancer testis or cancer/germ-line antigen genes expression [39]. Shinkai and colleagues reported that MAGE-A2 expression was activated in knockout HMT G9a^−/−^ *in vitro* and *in vivo* murine embryonic stem cells model [40, 41]. It was also shown that chaetocin produces minimal toxicity at concentrations necessary for peak induction [42]. Similarly, our previous study observed that the pretreatment of myeloma cells with JSI-124 and bortezomib can recover DC functions through the up-regulation of HSP90 and down-regulation of p-STAT3 and inhibitory cytokines, and that these DCs can potently generate myeloma-specific CTLs [15]. Therefore, we speculated that the induction of dying myeloma cells with the HMT inhibitor chaetocin acted to potentiate the expression of CTA and HSP90 on the surface of dying tumor cells. Treatment with chaetocin markedly induced the expression of HSP90, MAGE-A3 and MAGE-C1/CT7 on dying myeloma cells. The enhancement of HSP90, MAGE-A3 and MAGE-C1/CT7 levels by chaetocin, compared with UVB irradiation, was more evident on DCs loaded with dying tumor cells.

For an effective DC vaccine, DCs should have the potency to stimulate T cells and migrate through lymphatic vessels to interact with T cells. In this study, we demonstrated that DCs loaded with chaetocin-treated dying myeloma cells were recovered by increasing the expression of maturation molecules and producing low levels of the inhibitory cytokine IL-10 compared with DCs loaded with UVB-irradiated dying myeloma cells. Thus, we considered that the use of chaetocin may enhance the expression of the endoplasmic reticulum translocon protein Sec61 in DCs loaded with dying myeloma cells. However, the effects of chaetocin were abrogated by geldanamycin, suggesting that enhanced cross-presentation in these experiments was largely HSP dependent. Furthermore, DCs loaded with chaetocin-treated dying myeloma cells most potently inhibited CD25^+^Foxp3^+^ T cells, increased the proportion of CD8^+^ T cells, induced Th1 polarization and generated the most potent myeloma-specific CTLs compared with DCs loaded with UVB-irradiated dying myeloma cells.

In conclusion, our data suggest that pretreatment of myeloma cells with chaetocin can recover DC dysfunction upon loading with dying myeloma cells through the upregulation of HSP90 and CTA. These DCs can generate potent myeloma-specific CTLs.

## MATERIALS AND METHODS

### Generation of DCs from patients with MM

All experiments were performed after obtaining informed consent from the subjects, according to a protocol approved by the Chonnam National University Hwasun Hospotal Institutional Review Board. Monocytes were isolated from peripheral blood mononuclear cells (PBMCs) obtained from patients with MM (IgG, λ-type) using a CD14^+^ magnetic activating cell sorting (MACs) system (Miltenyi Biotec Inc., Auburn, CA, USA). Monocytes were then cultured at 5 × 10^5^ cells/well in six-well plates (BD Falcon, San Jose, CA, USA) in RPMI-1640 (Gibco-BRL; Grand Island, NY, USA) supplemented with 10% heat-inactivated fetal bovine serum (FBS, Hyclone; Logan, UT, USA) and 1% penicillin-streptomycin (PS) in the presence of 50 ng/mL of recombinant human granulocyte macrophage-colony stimulating factor (rhGM-CSF, LG Biochemical; Daejon, Republic of Korea) and 20 ng/mL of recombinant human IL-4 (rhIL-4, R&D Systems; Minneapolis, MN, USA). On days 2 and 4, the medium and cytokines were replaced with fresh complete medium-containing cytokines. On day 6, imDCs were harvested. Mature DCs were then generated by a further 48-h cultivation of 2 × 10^5^ of imDCs with 200 ng/mL of lipopolysaccharide (LPS) from *Escherichia coli* (Sigma-Aldrich, St. Louis, MO, USA) in 24-well plates (BD Falcon). The maturing DCs were loaded with dying tumor cells of the U266 myeloma cell line (HLA-A0201^+^ myeloma cell lines, λ-type) at a ratio of 2:1, 2 h after the addition of the LPS used for maturation.

### Preparation of dying U266 cells induced by chaetocin as a source of tumor antigen

On the day of the experiment, U266 cells were harvested and washed two times with RPMI 1640 (Gibco-BRL). The cells were induced apoptosis by seeded in the wells of 24-well culture plates in RPMI-1640 medium (Gibco-BRL) supplemented with 10% (v/v) FBS (Gibco-BRL) and 1% (w/v) PS, in the presence of chaetocin (Sigma-Aldrich) in a dose-dependent manner (25 to 400 nM) for 24 hours, or by high-dose UVB irradiation (120 mJ/cm^2^) (International light, Newburyport, MA) followed by overnight culture in RPMI-1640 (Gibco-BRL). Cell apoptosis was detected by an Annexin-V fluorescein isothiocyanate (FITC) and propidium iodide (PI) apoptosis detection kit according to the manufacturer's protocol (BD Biosciences, Franklin Lakes, NJ, USA). Next, the samples were acquired on a FACS Calibur cell sorter (Becton Dickinson, Mountain View, CA, USA), and the data were analyzed using WinMDI software (ver. 2.9; Biology Software Net: http://en.bio-soft.net/other/WinMDI.html). The cells were washed extensively before loading onto DCs at a ratio of 2:1 (DCs to dying tumor cells). In addition, the expression of inducible HSP90 in dying tumor cells treated with chaetocin was analyzed by flow cytometry using specific monoclonal antibodies (mAbs) for HSP90 (Stressgen, Ann Arbor, MI, USA).

### Quantitative reverse transcription-polymerase chain reaction

Total RNA was extracted from U266 myeloma cells with TRIzol reagent (Invitrogen, Waltham, MA, USA) and reverse transcribed using the first-strand cDNA synthesis kit (RT0212-03; Biomiga, San Diego, USA) according to the manufacturer's protocol. Quantitative reverse transcription-polymerase chain reaction (qRT-PCR) was performed with the Light-cycler 480 PCR apparatus (Hoffman-La Roche Ltd, Basel, Switzerland) using SYBR Green PCR master mix (DRR014A; Takara Biotechnology, Dalian, People's Republic of China). PCR reactions were performed under the following conditions: 95°C for 30 seconds, 40 cycles of 95°C for 5 seconds, and 60°C for 30 seconds. The relative levels of gene expression were calculated by the −ΔΔCt method using β-actin as a control and expressed as 2^−ΔΔCt^. PCR primers were used as follows: MAGE-A3: forward, 5′-GAAGCCGGCCCAGGCTCG-3′ and reverse, 5′-GGAGTCCTCATAGGATTGGCT-3′; *MAGE-C1/CT7*: forward, 5′-ACATCCTCACCCTCAGGAGGG-3′ and reverse, 5′-GACGAGGATCGTCTCAGGTCAGC-3′; *β-actin*: forward, 5′-ACCCACACTGTGCCCATCTAC-3′ and reverse, 5′-TCGGTGAGGATCTCATGAGGTA-3′.

### Western blot analysis

DCs unloaded with dying U266 cells and DCs loaded with UVB-irradiated or chaetocin-treated U266 cells were used to investigate the expression of the endoplasmic reticulum translocon protein Sec61. Total cellular proteins in the cell extracts were separated by 12% sodium dodecyl sulfate-polyacrylamide gel electrophoresis and were transferred to a nitrocellulose membrane. The membrane was incubated with 5% nonfat milk in phosphate-buffered saline (PBS) and incubated with anti-β-actin (Santa Cruz Biotechnology, Danvers, MA, USA), anti-Sec61A (Abcam, Cambridge, UK) overnight at 4°C according to the manufacturer's instructions. After washing, each membrane was incubated with horseradish peroxidase-conjugated goat anti-rabbit-IgG (Santa Cruz Biotechnology) for 1 hour at room temperature. Immunoreactive bands were detected using the WEST-one Western Blot Detection System (iNtRON Biotechnology, Daejeon, Korea) and were analyzed with the LAS-3000 imaging system (Fujifilm, Tokyo, Japan).

### Phenotype analysis

The mAbs against human CD80-PE, CD86-FITC, CD83-FITC, and CD40-FITC (BD Bioscience, San Jose, CA, USA) were used to detect surface markers on DCs using flow cytometry (FACS). Cell debris was eliminated by forward and side scatter gating. The samples were acquired on a FACS Calibur cell sorter (Becton Dickinson), and the data were analyzed using WinMDI.

### IL-12p70 and IL-10 production

DCs unloaded with dying U266 cells, DCs loaded with UVB-irradiated dying U266 cells, or DCs loaded with chaetocin-treated dying U266 cells were cultured in 96-well plates at 2 × 10^4^ cells/well and stimulated with CD40Ligand (CD40L)-transfected J558 cells (as an analog of CD40L-expressing Th cells; a kind gift from Dr. P. Lane, University of Birmingham, United Kingdom) at 5 × 10^4^ cells/well. After 24 h, the supernatants were collected, and the production of IL-12p70 and IL-10 was determined by BD OptEIA enzyme-linked immunosorbent assay (ELISA; BD Biosciences). Each sample was analyzed in triplicate, and the mean absorbance for each set of standards and samples was calculated.

### Polarization of naïve CD4^+^ T cells

2 × 10^4^ cells of DCs unloaded with dying U266 cells, DCs loaded with UVB-irradiated dying U266 cells, or DCs loaded with chaetocin treated dying U266 cells were cocultured with allogeneic CD4^+^ T cells (2 × 10^4^ cells) isolated from the PBMCs of healthy donors using the MACS system (Miltenyi Biotec) in RPMI-1640 medium containing 10% FBS and 1% PS. On day 5, rhIL-2 (10 U/mL; R&D Systems) was added, and the medium was replenished with cytokines every 2 days for 6 days. On day 11, T cells were harvested and re-stimulated with 1 μg/mL of CD3/CD28 mAb for 24 hours. The harvested cells were fixed, and permeabilized for intracellular IL-4 and IFN-γ production. To detect regulatory T cells, polarized cells were surface stained with an mAb against CD25-FITC (BD Biosciences) and subsequently stained with an mAb against FoxP3-PE (BD Biosciences), following the protocol of FACs permeabilizing solution (BD Biosciences) and using the FACS Calibur cell sorter (Becton Dickinson). The data were analyzed using WinMDI.

### Induction of myeloma-specific CTLs

2 × 10^5^ cells of DCs unloaded with dying U266 cells, DCs loaded with UVB-irradiated dying U266 cells, or DCs loaded with chaetocin-treated dying U266 cells were cocultured with 1 × 10^6^ cells of autologous CD3^+^ T cells (purity > 90%) obtained from the lymphocytes fraction of the same patients. On day 3, recombinant human IL-2 (25 ng/mL; R&D Systems) and IL-7 (10 ng/mL; R&D Systems) were added. These autologus T cells were restimulated with the same DCs on day 10. Finally, on day 20, the number of antigen-specific T cells was analyzed by the interferon (IFN)-γ enzyme-linked immunospot (ELISPOT) assay (BD Bioscience). The frequency of antigen-specific CTL lines was analyzed using U266 tumor cells as target cells with or without anti-MHC class I- and II-specific mAbs (20 mg/mL; clone W6/32 and clone CR3/43, respectively). The ELISOPT data were expressed as the mean (±SD) number of spots per 1 × 10^5^ T cells. CTL cells alone were used for the control group. The proportions of CD4^+^ and CD8^+^ T cells in the stimulated autologous T cells were measured by FACS Calibur cell sorter (Becton Dickinson), and the data were analyzed using WinMDI.

### Statistical analysis

All statistical analyses were performed with SPSS for Windows (ver. 13.0; SPSS Inc., Chicago, IL, USA). The Mann–Whitney *U* test was performed to analyze the statistical significance of nonparametric differences between the groups; a p value less than 0.05 was deemed to be statistically significant.

## SUPPLEMENTARY MATERIALS FIGURES AND TABLES


