# Supplementary material for: Chaetocin enhances dendritic cell function via the induction of heat shock protein and cancer testis antigens in myeloma cells
Source: Oncotarget. 2017 Apr 29;8(28):46047–56. doi: 10.18632/oncotarget.17517 (PMC5542247; doi:10.18632/oncotarget.17517)
Supplement: Supplementary file 1 [file oncotarget-08-46047-s001.pdf]

Chaetocin enhances dendritic cell function via the induction of heat shock protein and cancer testis antigens in myeloma cells

SUPPLEMENTARY FIGURES

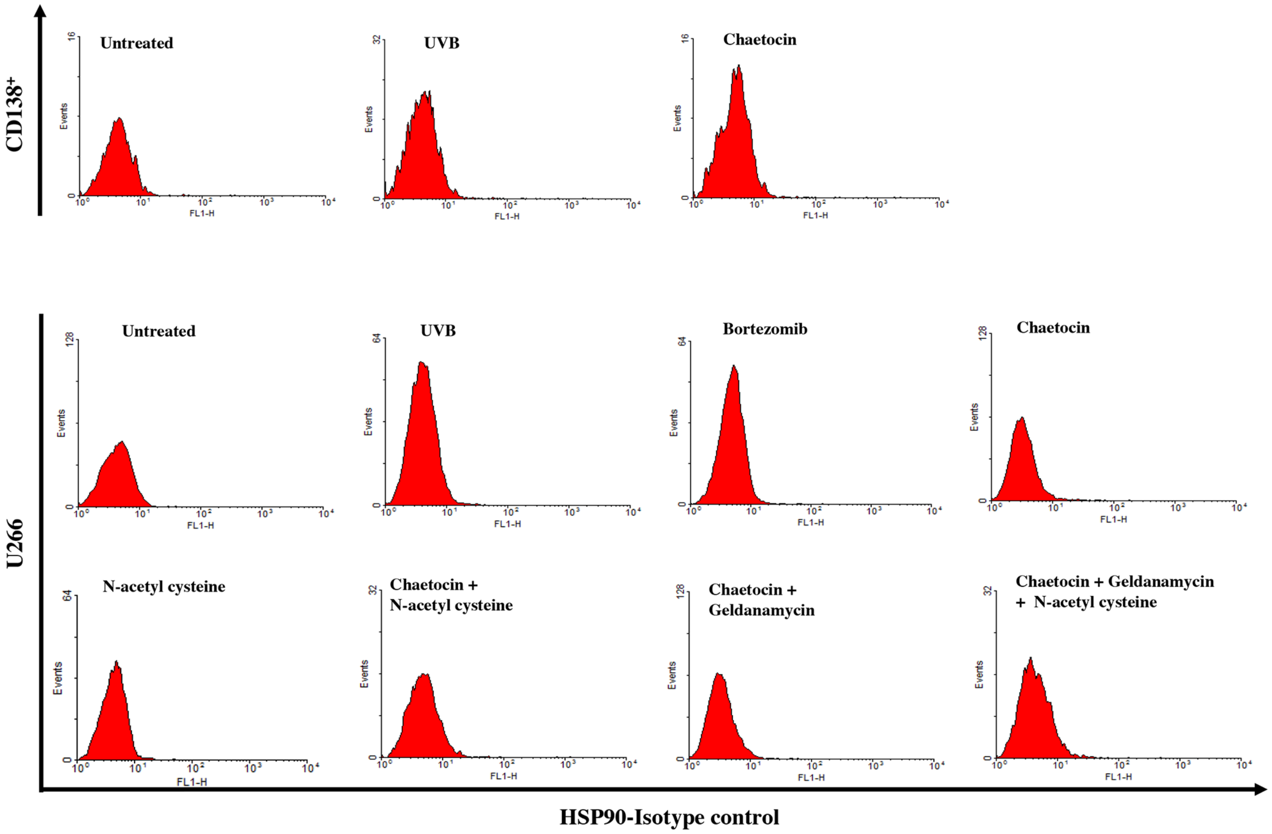

Supplementary Figure 1: Expression of HSP90 isotype controls FITC-labeled mAb in dying myeloma cells. The histograms represent levels of HSP90 isotype controls-FITC expression in dying myeloma cells (U266 cell lines and $CD138^+$ cells from patients). HSP90 isotype control FITC-labeled mAb expression was analyzed by flow cytometry.

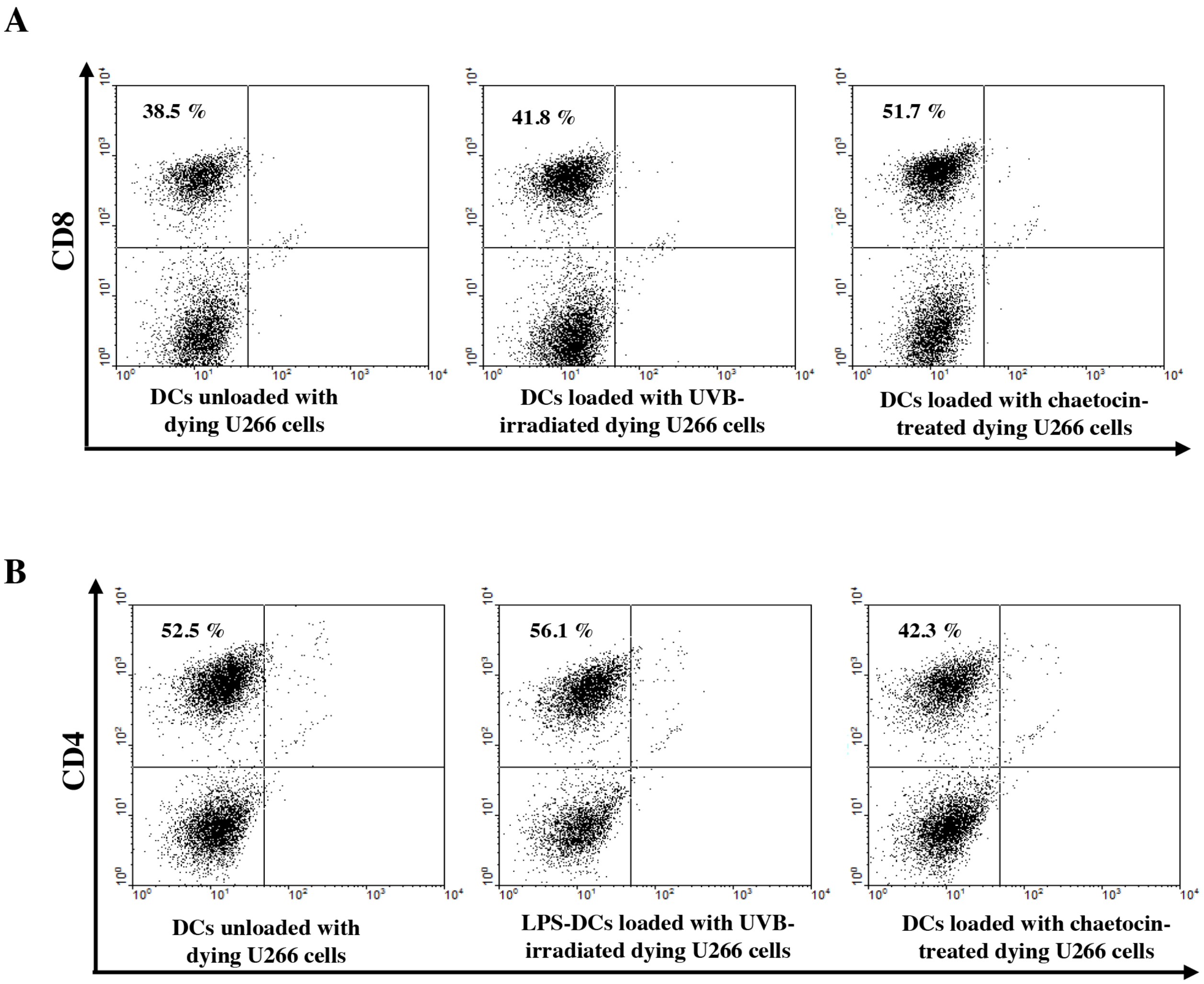

Supplementary Figure 2: The proportions of $CD4^+$ and $CD8^+$ T cells in the stimulated autologous T cells induced by DCs loaded with dying U266 cells. Autologous T cells (1×10^6 cells) were stimulated by autologous DCs (2×10^5 cells) loaded with dying U266 myeloma cells. On day 20, the proportions of $CD8^+$ T cells **(A)** and $CD4^+$ T **(B)** cells were measured using flow cytometry. DCs loaded with dying U266 cells treated with chaetocin led to an increased proportion of $CD8^+$ T cell and a decreased proportion of $CD4^+$ T cell compared with DCs unloaded with dying U266 cells, or DCs loaded with dying U266 cells treated with UVB irradiation. Data are representative of more than three experiments.
